# Disembodied creativity in generative AI: *prima facie* challenges and limitations of prompting in creative practice

**DOI:** 10.3389/frai.2025.1651354

**Published:** 2025-08-14

**Authors:** David Casacuberta, Ariel Guersenzvaig

**Affiliations:** ^1^Department of Philosophy, Universitat Autonoma de Barcelona, Barcelona, Spain; ^2^Elisava School of Design and Engineering, University of Vic-UCC, Barcelona, Spain

**Keywords:** creativity, generative AI (GenAI), design, arts, expertise

## Abstract

This paper examines some *prima facie* challenges of using natural language prompting in Generative AI (GenAI) for creative practices in design and the arts. While GenAI is purported to “democratize” creativity by offering a new mode of creation, we argue that it comes with a significant mortgage—particularly one in relation to expert performance, skill acquisition, and embodied engagement. Drawing from Dreyfus and Dreyfus, we show that creativity grounded in internalized expert knowledge cannot be reduced to rule-following or meaningfully externalized in instructions, i.e., prompts. Building on Polanyi, Simon, and Sennett, we posit that much of what makes creative work meaningful is tacit and intuitive, and therefore cannot be fully articulated through prompts. From the perspective of embodied and enactive cognition (Thompson, Noë, Pallasmaa), we argue that even “traditional” digital tools retain a material, bodily interface—something entirely absent from prompt-centered creation. While it may be tempting to treat GenAI systems as mere instruments, the mode of interaction they afford introduces a discontinuity: unlike analog tools or conventional software, they offer the creator significantly less control and disrupt and even erode the feedback loop between mind, hand, and expressive material. Rather than supporting skill development, prompting risks sequestering the user in novice-level engagement. By addressing these challenges, our analysis offers a clearer view of what is at stake when generative systems are integrated into creative disciplines, and why human creators, integrating multiple creative and epistemic faculties as they see fit, must remain at the center of that process.

## 1 Introduction

All creative processes involve a dialogic interaction between the creator and the artefact being developed—be it a painting, a novel, an interface, or a philosophical essay. This “back-and-forth” dynamic is mediated through tools, ranging from simple ones like paper and pencil to sophisticated software. Every tool enables the creator to move in particular directions according to its affordances, but not in all directions: one cannot use a pencil to carve marble. However, in principle, the tool itself, though it can greatly condition both the process and the outcome, does not fundamentally change the ontological nature of the creative act.

One could argue, as Shen et al. (2025) do, that generative AI functions in the same way as just another technological mediator. For instance, creating an illustration with paper and pencil involves iterative interaction with those tools and the act of drawing, and the same can be said of generative AI; only, in this case, the tool consists of the prompts we type into the dialogue box. Therefore, using prompts in an AI program would be a change only in the properties of the mediator, while still being a fully creative act, not ontologically different, for instance, from a paper-and-pencil-based creation.

Following that train of thought, we can say that a typeface can be designed using different sorts of tools, such as analog tools like quill pens, software tools like FontLab or Robofont, or we could utilize a large language model specialized in typography to generate it with detailed prompts that capture the main properties of the desired font. But in the end, what we get is a human person creating a typography that other people can use in their own creations. The tool would not really matter.

But is that really the case?

In this paper, we argue that using prompts with GenAI systems differs in essential ways from working with analog tools or “conventional” digital tools because certain aspects of human creativity resist codification into linguistic instructions, i.e., prompts. To flesh this out, we begin in Section 1 by analyzing the nature of the interaction between humans and large language models (LLMs). In Section 2, we will present basic concepts on human creativity, drawing on ideas from philosophers such as Dreyfus and Dreyfus, and Polanyi, cognitive scientists like Simon, and design theorists like Maeda, to establish how human creativity processes differ from instruction-only approaches. In Section 3, we confront ideas from the first two sections to highlight the main differences between human creativity and the processes facilitated by generative AI algorithms. Section 4 argues against the idea that even if different from human creativity, such a new way of creativity is actually better than the human one, introducing some closing remarks in Section 5.

### 1.1 Modes of existence of LLMs

How do large language models participate in creative processes?

To answer such a question, we need, first of all, a better understanding of the nature of large language models and how they relate to the designing or artistic products they can generate. Following Simondon’s (2011) ideas on the philosophy of technology and their modes of existence, we can ask ourselves about the mode of existence of a large language model.

Opinions clearly vary from amazingly inflationary to pure deflationary (Mitchell and Krakauer, 2023). Some views defend the idea that they are fully autonomous agents that meet the criteria to be considered scientific contributors (Miller, 2023) or even conscious creatures equivalent to humans (Lemoine, 2022), in contrast to the perspective that they are merely stochastic parrots (Bender et al., 2021).

Marcus (2024) presents large language models as enormous look-up tables that resemble Borges’s (1994) “The Library of Babel”, a story about an infinite library containing every possible combination of letters in books of a fixed format, which therefore holds not only all meaningful texts ever written but also every possible nonsensical variation. In Marcus’ tables, in the end, you cannot expect any authentically new results, just combinations of the data that is already in one of these tables that the system looks up. However, if tables are large enough to contain millions of data points and can be reshuffled and combined, then does it still make sense to call them just stochastic parrots?

To clarify the mode of existence of large language models, we will draw on Barandiaran and Almendros (2024) and Barandiaran and Pérez-Verdugo (2025), who examine, among other things, the role of large language models in the creative process. They observe that due to their disembodied and pure linguistic nature, large language models cannot be considered fully functioning autonomous agents.

More specifically, using criteria borrowed from the enactivist paradigm, they observe that large language models do not meet the conditions for autonomous agency. In particular, LLMs fail to meet the individuality condition, which requires the product to be the result of its own activity. They also observe that the normativity condition is not met because the system does not generate its own norms or goals, but is fully dependent on those formulated by the human being who uses the program. The interactional asymmetric condition in which the system asymmetrically regulates its coupling with the environment thus becoming a source of action is only accomplished partially (Barandiaran and Almendros, 2024, pp. 18–21).

However, Barandiaran and Almendros (2024) do not view them as mere stochastic parrots, so they argue that large language models like ChatGPT^1^ should be characterized as a sort of linguistic automaton. They present the apt metaphor of an interactive library, or, in their own words, a “library that talks.” That is, those systems are not truly autonomous agents, but they can help co-create processes and objects through an interactive and iterative set of tasks.

Due to their inherent nature as a lookup table and the architectural constraints, the system may not always have the answers or the expected sought objects. Still, it is impelled to find them because an output must be generated, so it will inevitably “hallucinate”; that is, generate an output that is factually incorrect or outright absurd. However, for the sake of this paper’s aim, we have set aside hallucinations. Neither do we discuss whether these systems are “bullshit machines” (Hicks et al., 2024) or if this label is apt (Gunkel and Coghlan, 2025). We are comparing creative processes, so the truth value of the results is not our concern, but rather whether they are helpful for creators to develop their own projects.

Barandiaran and Almendros (2024) and Barandiaran and Pérez-Verdugo (2025) argue that this new type of co-creating stuff based on large language models goes beyond the type of instrumental creation that we have seen so far with digital tools (like InDesign or Photoshop). They call it a “midtended” form of agency that is closer to what an intentional agent will make than a pure instrumental approach, such as using the mouse to edit a picture in Photoshop. Specifically, they refer to “generative midtended cognition” as a new variant of “extended cognition,” which captures “a space situated between traditional conceptions of intention or intended creation, that is, generated from within, and extended, processes that bring material exo-biological processes into the creative process” (Barandiaran and Pérez-Verdugo, 2025).

Barandiaran and Almendros (2024) seem to argue that this full combination of human talents and digitized human knowledge from the talking library brings about a new creative process that is something completely different from previous ones. Therefore, they are not arguing that creative human intention is equivalent to prompt-based creation. Instead, they say that prompt-based creation is something different—a new type of creation.^2^ We agree that these are different. Still, we will say that several issues undermine this new type of creativity—or “generative midtended cognition” in Barandiaran and Almendros’s (2024) terminology—which does not seem like a suitable or equivalent substitute for the human interaction of back-and-forth among the technology, the person creating, and the media used to shape the creation, such as a hand with paper and pencils.

### 1.2 Being *inspired by* is not the same as *creating with*

It is important to distinguish between using LLMs as a source of inspiration or direction versus using an LLM as something to create the actual artefact *with*. In the first case, prompts are used to generate ideas that a person can further develop without an LLM. To illustrate, a result that can serve as inspiration would be something like: *Draw a city skyline at sunset using only geometric shapes and a limited color palette to explore the relationship between structure and atmosphere.*

Despite what Barandiaran and Almendros’s (2024) say about a certain intentionality in creation through prompts—what they call “midtended” form of agency—if the use of large-language models is mostly about inspiration, and human beings are the ones that make the final creative object, then, we posit, we are still talking about something that a human person creates through the use of an instrument.

In the *creating-with* case, prompting itself becomes the core creative mechanism. The final product is the result of selecting and minimally editing AI outputs. We term this “the GenAI stand-alone mode of creation.” In this case the result of the prompt would be the *actual* illustration of a city skyline at sunset.

Based on what we have argued so far, we can characterize such a stand-alone mode of creation as a pure linguistic and declarative way of creativity. Therefore, a model that is disembodied, based on instruction-driven outputs, fully dependent on large datasets, and presenting a degree of randomness inherent to probabilistic generation.

How close is this stand-alone mode to human creativity? Not so much. In order to show this, we will explore in the next section what the mode of existence of human creativity is.

## 2 Human creation and creative cognition

### 2.1 Creativity is an embodied process

From McLuhan (1964) to Pallasmaa (2017), a consistent body of literature has defended the key relevance of the hand as the interface between the creative product and the technology used to build the cultural or artistic object. In the stand-alone mode of creativity offered by generative artificial intelligence, the role of the human hand is conspicuously missing. What we have instead is text production through typing on the dialogue box or dictating prompts via a microphone, so there is no hand interface at all.

Creativity is also linked to abduction. Abduction, as a form of logical inference, is the process of generating the *best explanation* for a set of observations. Unlike deduction, which moves from general rules to specific conclusions, or induction, which generalizes from specific instances, abduction proposes a hypothesis that, if true, would account for the observed phenomena. In an embodied context, this is not a purely linguistic or symbolic process but is deeply rooted in our sensory and motor interactions with the world. The “gut feeling” or intuition described in the context of expert knowledge, for instance, can be seen as an embodied form of abductive reasoning, where years of experience allow a designer or architect to implicitly generate plausible explanations for perceived problems or potential solutions, even before they can articulate them verbally. Abduction, therefore, is a key element in any creative process.

Furthermore, abduction is not an individual process: Our abductive inferences are not contained solely within the brain but are distributed across and deeply intertwined with the external world. In creative practice, this means that the artist’s adoption of a novel artistic direction is not just an internal flash of insight but emerges from the active exploration of materials, interaction with tools, and the subtle cues perceived in the surrounding environment. The “thinking hand” described by Pallasmaa (2009, 2017), for example, embodies this eco-cognitive openness, where the hand acts as an interface that not only executes but also participates in the generation of ideas, allowing for an ongoing abductive dialogue between the creator’s intentions, the material’s affordances, and the evolving artistic product (see Magnani, 2021, 2022).

Creative texts are not the target of this essay, but rather visual products that belong to design, architecture, or the arts. However, the fact that the hand is not so thoroughly implied in creating fictions or essays does not mean that GenAI’s stand-alone creativity and human creativity are equivalent when creating original text.

One could argue the opposite because, in the end, text linguistic corpora are the only medium implied. However, we want to argue that this is not the case. Writing a scientific paper, or a poem is an iterative and embodied process of a back and forth between the text on a screen or a sheet of paper and the aims and ideas of the human author, reading what the author has written so far, revising it in their own mind, making changes to the text, and then revising it again.

Instead, think of the novelist as providing a lengthy prompt to generate a new chapter in their book. It may be something like this: “In this chapter, we are going to describe how characters so-and-so interact in this situation, leading to such and such things to happen, and so on.” Then, the person hits the return key, and the language model generates a full chapter. This would be a GenAI stand-alone creation of a text, and it will work as Barandiaran and Almendros’s (2024) describe; that is, as a midtended process. However, such a process does not resemble writing a novel. It mostly resembles a busy politician hiring a ghostwriter: The politician provides the writer with some highlights of his life, and then the ghostwriter turns them into a book of memories.

That being said, let us consider visual creation by a GenAI stand-alone mode. Can we really compare it to humans creating photographic works, designing book covers, or making animations?

Pallasmaa (2017) argues that the key role of the hand is to serve as an interface between the body and the mind. So, when an architect designs a building with paper and pencil, the hand serves as a bridge that turns thoughts in the architect’s mind into a design—a floorplan or 3-D model of the building. Through this process, the hand is a two-directional interface between thoughts and the final images. However, and this is key, the thoughts are not merely linguistic thoughts that one can simply turn into prompts. The tools (whether a pencil or a software program like AutoCad) are not just mere instruments: they are extensions of the designer, artist, or architect’s bodies connected by their hands. In this way, every designer, artist, or architect uses their instruments differently, leaving its own mark even when using the same tools. Both Picasso and Braque used oil paint and even painted similar subjects. Yet their results are unique. Heidegger and von Herrmann (1977) argue that when one is fixing a wooden roof with a hammer, that person thinks with the hammer in hand, so the hammer is an extension of the body and the mind of the person, and they are all together in touch, making a unity that is bigger than the parts. This explains why Picasso and Braque, both Cubists even, left distinct marks on their paintings despite using the same paint.

Following ideas of the enactivist paradigm from Thompson (2010) and Noë (2004), we can say that the creative process is something that is distributed between the mind of the creator in a classical sense, the body of the creator (especially their hands), and the surroundings that give clues on how to act. The three elements are all important: the thought is situated in the body and the specific surroundings, and again, the surroundings are non-linguistic. They are not just text-based datasets like those used to train a large language model. We are talking here about living surroundings that encompass nature and other technologies, which are themselves an extension of the body. So we have this beautiful loop between body, mind, and surroundings that together create something. Importantly, as Pallasmaa (2009) posits, in this loop, the body and the results we are seeking are intertwined: we would not use our hands in a meaningful way without being able to imagine the outcome of our action. In this sense, the technical knowledge of a designer, of an artist, of an architect is not just linguistic data. It is expert knowledge that comes from touch, from the hand that belongs to the body and interacts with technology and cultural objects, as well as with the intended purposes, however explicit or tacit they might be.

Let us consider drawing, for example. In drawing, several senses are activated together (at least sight and touch of the pencil, its friction on the paper, and what the pencil draws on it). Also, when an architect plans the structure of a building or a product designer imagines a new coffee machine, we are not talking just about the linguistic knowledge acquired by reading design or architecture books. We are referring to physical models that reside in the architect’s mind and body, which enable the exploration of material properties, shapes, textures, and surfaces that cannot be expressed purely in linguistic terms. So, when a creative person draws, this act of drawing is the result of the integration of senses, movements, and thoughts.

As we’ll see in the next section, when a expert draws, it is different from what a novice does. Here, this unity of senses, body, and mind acts like a subcognitive unity—purely integrated and not needing consciousness, appealing to rules to develop a creation. Knowledge like this is what enables the creator to see, or more accurately, to intuit problems and solutions. That is the common process in which an architect, an artist, or a designer senses that something is wrong or that something is right, but they are unable to express it linguistically. They just have this gut feeling about that, which more often than not cannot be expressed in words because it is not linguistic-declarative knowledge but tacit knowledge that is based on experiences that are based on having a body that relates to real objects in the real world. We are talking here, for example, about tactile memory and muscle memory that are not just accessible to our linguistic demand. Also noteworthy are myriad mental phenomena such as value judgments and preferences that might, at least initially, escape declarative knowledge.

All of this is clearly illustrated in a scene from the documentary *Sketches of Frank Gehry* (Pollack, 2007), about the Canadian-American architect Frank Gehry. In this scene, Gehry is upset, unsatisfied with a model for a building he is working on. Gehry says to his model maker, “Let us look at it for a while. Be irritated by it. Then we’ll figure out what to do.” Sidney Pollack, the documentary’s director, notices Gehry’s discomfort and asks him, “What don’t you like?” Gehry’s candid reply is, “I don’t know yet. It seems a little pompous, a little pretentious. There’s a part of it I do not know how to put in words.” Gehry’s dislike was ostensibly and unambiguously expressed somatically, but lacked, at least initially, a declarative explanation, which was later produced, but only tentatively.

Richard Sennett (2008) also defends an embodied approach to creation, suggesting that knowing a craft is the result of exercising that craft to make things, and that touch is, in itself, a way of thinking. Almost all the knowledge of a craftsperson is based on bodily practices.

The way one learns a craft is by working repeatedly on the same tasks, and one does not reach mastery until one is able to turn those practices into full intuitions arising from internalized knowledge. That knowledge is gained through practicing and making mistakes in the process. However, those mistakes are not just problems to be solved but ways of getting feedback and improving our craft. Sennett also defines the craftsperson as someone who is motivated by full autonomy. They are interested in craftsmanship for its own sake. They are not looking for any external reward—money, fame, or status. They want to do a good job for ethical reasons. While endorsing the idea of craftsmanship as “autotelic”—becoming an end in itself—some authors (notably Korn, 2013) have called this *idealistic*. Be that as it may, it seems clear that such a type of motivation is not going to be found in a standalone generative AI process for obvious reasons, and we have argued above, using Barandiaran and Almendros (2024), that a stand-alone GenAI creative cannot be considered an autonomous agent.

### 2.2 Creativity beyond discourse

In order to properly understand what creation is, we need to avoid the idea of only one type of creator. This “one size fits all” approach when talking about artistic, design, or scientific creators does not work. We need to distinguish clearly between the novice who is starting to learn a new craft and the expert, the professional who has been designing computers, book covers, or buildings for years and has access to internalized, tacit expert knowledge. In Dreyfus and Dreyfus (1991), we are presented with an analysis of the learning process to become an expert in something, which is divided into five stages of the development of expertise. This five-stage model starts at the novice level. In this level, the person who is beginning to learn is completely dominated by rules and plans, so their process is almost completely declarative, and they needs access to rules and declarative knowledge in a conscious way to operate properly. Consider a novice starting to learn chess. This person has to stop and remember how the knight moves and how it is different from the way the queen moves, and revise that in order to decide what is the best move. They might also consider context-less rules such as “use all your pieces” or “protect your king.”

Then we move to the next step, the advanced beginner. This person still has to access those general rules but is also able to decide which rules are more relevant and why, depending on the conditions around, but they still have to identify in a conscious way the specific situation they are in. Continuing with the chess example, an advanced novice examines his opponent’s last movement, and after some revision of the rules, observes that their opponent moved the rook in order to capture an unprotected pawn. Then they have to think consciously about the best way to protect a pawn.

Next, we have the third stage, called Competence. At this moment in the learning process, the person realizes that there are too many rules to access them all and starts to organize the materials around organizing principles, so they can decide which information is relevant and which actions are more useful. Still, one must make conscious plans and follow specific procedures.

In the fourth stage, the Proficiency stage, the performer is finally able to see holistically. They do not need conscious recognition of a mistake in their creation to realize that something is wrong and then subconsciously act to solve it. In the chess example, the player sees an opportunity to gain some material, sensing that their opponent has made a wrong move, and a winning move comes to their mind directly. In Gehry’s documentary, Gehry senses something is wrong *before* knowing what is wrong, He seeks to provide an explanation of what is wrong only when pressed by Pollack.

In the final level, the Expertise level, the expert does not rely on rules anymore and uses their intuition to make decisions. Planning, acting, and diagnosing are mostly done without any analytical calculations; they just flow naturally from this embodied, intuitive, tacit understanding of the problem. We could say that in this stage, the expert no longer makes decisions consciously or solves problems consciously; instead, it is more of a “what must be done” situation—it is simply done. Analysis is only performed in cases where the situation is significantly different from usual or when additional clarification is required. To illustrate, in another scene in Gehry’s documentary, he is, again, visibly stuck and not happy with another part of the model. All of a sudden, Gehry says, “I know how to do it. Just corrugate [the cardboard]” (Pollack, 2007). Then his model maker modifies the model, and Gehry is pleased with the result: “See how it works?”

Creating through prompts clearly does not fit within this understanding of the five stages of the creative person. A person who uses a standalone mode based fully on generative AI to produce illustrations will never move beyond the first stages, especially if this person has never been taught to draw with paper and pencil. Such a person is stuck in a rule-based system *by design*. They may have some intuition about how some prompts work better than others, but this is only limited intuition, and it is always based on understanding a set of rules because, at all times, that person is interacting with the system through linguistic instructions. The very design of prompt-based LLMs requires that the creator give explicit instructions, thus excluding the more sophisticated, intuitive, non-declarative skills belonging to the higher stages, which, to reiterate, do not require words.

Therefore, following Dreyfus and Dreyfus’ five-stage model of skill acquisition, we see that expert creativity cannot be reduced to discrete verbal instructions. The prompt is thus an insurmountable obstacle.

Another way to understand the specificity of expert knowledge is to observe how knowledge beyond the initial stages is primarily tacit knowledge. That is, we are talking about knowledge based on muscle memory, tactile memory, and knowledge that is the result of continuous familiarity with the material one is working with. Such a growing sense of familiarity leads, as Simon (1987) argues, towards a pure intuitive, subconscious understanding of the materials and procedures, so that it becomes tacit. And tacit knowledge, by its very nature cannot be fully turned into linguistic instructions (Polanyi, 1966). Sennett (2008) and Pallasmaa (2009, 2017) see the matter in a similar way.

Consider the classical example of learning to ride a bicycle. In order to learn to ride a bike, the only way is actually riding a bicycle, getting on a bicycle, trying to move around, falling, getting back on, and falling and getting back on again until 1 day it comes naturally to us. Instructions beyond “look at the front and keep on pedaling” are useful, but they only capture a very small fraction of what is necessary to ride a bicycle, and all this knowledge is tacit. “Try to maintain your balance” might be good encouragement, but it is not actionable knowledge.

The knowledge that is necessary to ride a bike cannot be transformed into linguistic knowledge, and the same can be said about drawing. In order to learn to draw, you have to draw and draw and draw until the hand naturally starts to draw without having to follow instructions. Of course a good instructor can offer guidelines, tips, and tricks, but these fully rely on the student’s expected failure, and on their disposition to progress in order for the student to learn. Like the case of the violin student’s mistakes discussed by Sennett (2008), instructors guide learners patiently, viewing failure as an opportunity to grow and improve rather than just an error to fix. But the instructions and the errors do not amount to learning. To learn, the student needs to do *a lot more* than failing.

In the beginning, a person learning to draw might find the typical schemas helpful, such as drawing circles in a specific way and following rules to make a portrait of a face. However, if one remains at that level, one will be merely a perpetual novice, creating faces that are culturally irrelevant because they are too basic and predictable.

To recapitulate, creative knowledge is tacit (Polanyi) and intuitive (Simon); therefore, creators will have great difficulties trying to convert such tacit and intuitive knowledge into instruction-like prompts.

### 2.3 Not all mediating technologies are equal

Another course of action when discussing human creativity vs. creativity enhanced by digital technologies would be to question the neutrality of digital technologies.

For example, John Maeda and other design scholars argue that most digital tools are not transparent to the user, in the way that a pencil is, so mediation between the user and the technology will be different, and the user will lose some control in the process (Maeda 2001; Reas and Fry, 2006). According to such authors, when designing a book cover using software like Illustrator, this is fundamentally different from designing with paper and pencil because the software automatically creates elements that the user cannot control. This is sometimes referred to as the “Postscript Autocracy.” Therefore, Maeda, and Reas and Fry argue that in order to really have full control of digital creation, one needs to learn to program it. That is the only way in which the creator has full control of the system.

But, in the end, what is programming? At its simplest, it boils down to specifying a set of very precise rules. It is a completely declarative exercise. So maybe using prompts is actually the best way to create within a digital environment.

However, such an argument does not hold. If one wants to be fully in control of the result of a digital creation, then learning a specific programming language is certainly better than using a digital tool that has certain behaviors that are not accessible to the person using digital technologies with a mouse in hand. This is clear. But using LLMs is not like programming at all. Actually, it implies including a huge collection of lookup tables of possible images in a bank of images that is actually thousands of times more autocratic than the worst visual design software. The same phenomenon is happening right now, as some computer science students are trusting LLMs to generate code instead of programming themselves, and are becoming significantly less proficient than those who write their own programs (Prather et al., 2024; Dou et al., 2024).

Nevertheless, Maeda’s distinction is relevant to understanding that there are different types of mediators. The best mediators are those that can be fully utilized by the creator, who understands what is happening and is able to think with the tool. This is certainly possible with analog tools that offer us almost full control beyond the materiality of the tools and the media we use. So, you cannot change the nature of paper or the nature of a 12B pencil, but that is all you have to worry about. The rest is completely up to you, and through diligent practice, you can master the art of drawing by hand. We can discuss whether Maeda is right, and whether programming gives you better control than using a digital tool. Probably the best solution is to design your own tools, giving you full control over what they do. The important thing is that, as Barandiaran and Almendros (2024) pointed out, there is a huge leap in the lack of control when we design and create using LLMs; it is not like programming at all. So we should not be fooled by the fact that programming and using a generative AI tool includes giving instructions; in programming, instructions are precise, and we can understand—if we want—the effect of the instruction at a pixel level. GenAI will give us none of that.

The leap between what we want and what the system generates can be so huge and so autocratic that not even the creators of the tool can tell us exactly what is going on.

## 3 What creativity is and is not

Still, one could say: Okay, so it is a different way of creating. However, it may be a more effective way of creating because it offers new opportunities that are currently unavailable to all users. So GenAI will give us a new type of creativity, more accessible, democratic, scalable, and better to promote “divergent” thinking (Eapen et al., 2023).

We can concede that it is a different way of creating, and we can agree that it might open up space for innovative ways of creation that are not currently accessible to authors. But how many of such innovative traits of GenAI stand-alone do we really need? Let us review what these new features could be and decide whether they are worth it or not.

### 3.1 Creativity is meritocratic, not democratic

Nowadays, users who do not have any knowledge or skills in drawing can nevertheless create astonishing illustrations in just seconds using generative artificial intelligence software. Some argue (e.g., Mark et al., 2023) that thanks to artificial intelligence, we are making creativity more democratic, so we can turn everybody into artists if they want to. But what type of artist? There is a fallacy hidden in this argument; primarily in that it establishes all creators are equal. But they are not. Returning to what we have said in Section 2.2 about the five stages of learning a craft, because generative AI is a stand-alone type of creativity that is rule-based, we will never move beyond the novice stage because we will always be thinking of explicit rules that we have to plan carefully. What we get comes down near to pure declarative generative systems, which are much more limited than human creativity as they disregard—because they cannot be included in the prompt—*all* the other ways of knowing and doing that are non-declarative! Needless to say, only what is explicit can be included in the prompt.

### 3.2 Visual creations are more than pixels

We should not forget that a cultural visual object has lots of emerging properties that cannot be captured in pixels. Ultimately, what generative artificial intelligence does to create images is a highly detailed and efficient analysis of statistical regularities in a dataset, but this is distinctly different from what a work of art or a design entails.

We can capture some of the creativity behind Picasso by seeing how his creations in his cubist period are statistically similar, so we can make cubist images close to Picasso, and we can also find similarities between his cubist paintings and cubist works from other authors. But then, the cultural milieu, the connection with Einstein’s theory of relativity, the inspiration that Picasso received from African art and so on, will be completely lost, so we are just tackling in a very superficial way the similarities between works of art, and we are missing the more relevant, the more interesting aspects of creativity for human beings. Similarly, the assertion we make when we say Picasso’s *Guernica* is “a great work of art” involves much more than the 3.49-m canvas covered with oil paint. Otherwise, we would say that a reproduction of *Guernica* is also a piece of art, but we do not. We say it is a “reproduction” of a work of art because we recognize the original’s artistic significance includes its historical context, inspiration, cultural significance, and creative intent—elements that a reproduction cannot possibly capture.

### 3.3 Creativity does not need to scale

A supposed benefit that has already been argued in several papers (e.g., O'Toole and Horvát, 2024; Haase and Pokutta, 2024), is scalability. An illustrator that is making images for a children’s story using human creativity and traditional analog or digital techniques will invest a lot of time and effort creating each illustration. In contrast, a generative AI system could scale that output, allowing the same artist to make 50 illustrations for the story (plus versions from the web and even animated videos) in just a tiny fraction of the time needed to create one illustration by hand.

So, how would that work? Imagine this illustrator having a huge database of all the illustrations they like from their preferred authors, organized by subject, type of content, background, and so on. They have developed a fine-tuned system in a large language model to create their own illustrations by utilizing subject headings, content types, and other relevant factors.

Problems would be inevitable: hallucinations, repetitive results, possible copyright infringements, and ultimately, realizing that their work has lost its edge, as it is through constraints and friction that real creativity works. Efficiency might be important in some cases but it is not the primary value of creativity; one could even wonder if it is a value at all. Actually, according to Sennett (2008), it would be a violation of the ethical principles and the ethics of a craftsperson. We should understand that scaling can be a very good thing in business, but it does not sound like a paramount demand or worry for human creativity.

### 3.4 Efficiency is not a key value in creative processes

Along these lines, the standalone approach to creation by generative artificial intelligence might be more efficient, but artists and creators are not particularly interested in efficiency for its own sake. It will mess up things ontologically because the key element of analysis is the pixel and the output, which are insufficient to understand creative processes and produce relevant cultural objects. In the end, what we get is a straightforward way to create based on patterns and function optimization to produce likely outcomes, and this inevitably leads to seeing the same things repeatedly. Scalability begets efficiency, and it is a lot easier to make illustrations, so the terrain is rapidly flooded by these predictable second-hand creations, which are devoid of meaning, context, and cultural significance.

The best way to understand that this is a dead-end path is to spend a couple of hours in Explore at an app like Sora, which generates video. We’ll see the same schemes, the same type of images, *ad nauseam*.

### 3.5 Automating creativity inevitably leads to cultural biases

While one browses through the images created by the users in apps like Sora, one will also see the same type of cinematic references, the same type of visual puns, the same type of faces, over and over and that leads to another big issue in automatic creativity: biases, which are a result of Generative AI’s dependence on training data and a lack of contextual understanding and intentionality.

AI-generated cultural and artistic creations are inevitably biased as they feed on finding and learning from patterns in their training datasets. Using “inclusive” or “curated” algorithms to avoid such biases is also a dead end, as we saw recently with the generated images of African-American Nazi soldiers or Native American women Nazi soldiers produced by generative artificial intelligence (Robertson, 2024). As we mentioned in Section 3.2, images are not just pixels, and one cannot determine the cultural and political principles and constraints of Nazi Germany solely by examining the pixels of pictures taken during that time. Naturally, the biases can be also in aesthetic or formal matters, as we alluded to above. Generative AI replicates the culturally dominant aesthetics, which limits creativity.

The only way to really find and reverse biases is through human creators who understand the existence of such biases from a cultural/social point of view and take decisive steps to remove them. A phrase like “astonishing beautiful sunset,” which is so boringly present in any image-like generator, is a term that is inevitably fraught with biases, and no matter how long your prompt is, you will not escape the bias because the bias is in the training corpus (Caliskan et al., 2017). The only way to escape the biased view of what a beautiful sunset is, is by cultivating the imagination, either by drawing it yourself or by encountering it, actively looking for the type of sunset one finds beautiful and taking pictures of it, or, if one is lucky enough, being caught off guard by it.

## 4 Discussion

### 4.1 Recapitulation

All the relevant information has been presented. So let us make a quick recapitulation now:

1. Drawing from Dreyfus and Dreyfus, we assert that human creativity cannot be reduced to rule-following or instruction parsing.
2. Drawing from Polanyi, Simon, and Sennett, we showed that because most creative knowledge is tacit and intuitive, creators cannot convert it into a prompt in a successful way.
3. From the enactivist perspective of Thompson and Noë, as well as the work of Pallasmaa, we argue that even digital tools retain an embodied component of creative interaction, which is lost in prompt-only workflows.
4. While both human creativity and GenAI outputs involve a form of contextual dependency, the embodied, situated nature of human creativity is qualitatively different from the pattern-trained, probabilistic responses of LLMs. The randomness of LLM “hallucinations” differs fundamentally from the situated, co-dependent, open-ended, embodied process that emerges through interaction with natural or social environments.
5. Even if one concedes Maeda’s point that most commercial digital tools constrain creativity, we maintain that GenAI introduces a discontinuity. Unlike analog tools or even conventional software interfaces, the prompt-based nature of GenAI systems offers much less control to the creator than ever before.

### 4.2 Let machines be machines, let humans be humans

Should we then suppress Generative Artificial Intelligence? Not at all. As we see in Section 1.1 when talking about the modes of existence of LLMs, they offer us a new tool, with midtended properties that humans can use as a new type of creative material (cf. Sangüesa and Guersenzvaig, 2019). Such an interaction is possible, but due to the way in which GenAI apps are marketed right now (e.g., as “text-to-video” or “text-to-illustration” tools), the temptations and incentives of using them as oracular machines that do *all the work* for us are so great that such an innovative and alternative way to create might be overlooked by users.

The deployment of GenAI products should then shift toward tools that provide creators with more transparent control over AI processes. A first and important step will be to move from big commercial models like ChatGPT or Google Gemini to local models, run on the users’ computers (Reddi, 2025). That way, key ethical concerns, such as privacy protection, environmental impacts, and workplace practices, as well as avoiding copyright infringements, are easier to address. Also, by fine-tuning on their own datasets, users can develop models that better reflect their social, ethical, aesthetic, and cultural values.

However, if we really want to put forward applications that are really in tune with all the idiosyncrasies of human creativity, we need to substitute the current dialogue box—turing test like interface—for more sophisticated interfaces, which shall include embodied type of interfaces where the hand and tacit knowledge can play an important role. There are some relevant extensions to current text-to-image models that are a promising direction to such a goal, like ControlNet (Zhang et al., 2023) or DragGan (Pan et al., 2023) which allow editing an AI generated image with the mouse.

## 5 Conclusion

Generative AI, at least in its current form, leads to a disembodied, instruction-dependent, data-driven, and inherently stochastic form of creativity. This makes it ontologically distinct from human creative practice. Rather than adopting what we call the GenAI stand-alone mode of creativity—i.e., treating these systems as autonomous creators—, we propose reimagining them as a new type of creative material with bounded agentic properties that assist, rather than replace, human ingenuity. This proposal certainly deserves further treatment, for which we now, alas, lack the space.

To achieve this, generative AI should shift toward tools that provide creators with more transparent control over AI processes. Moving from large-scale commercial models to locally hosted systems is a start, but more embodied, interactive interfaces are needed to fully integrate these tools into the human creative workflow.

The conceptual distinctions between human and GenAI creativity proposed in this paper have significant real-world applications for creative industries and education. By recognizing the disembodied, instruction-dependent nature of current GenAI, practitioners can avoid the pitfalls of a “stand-alone” mode of creation and instead present Generative AI as a tool that needs supervision and makes sense in some contexts but does not in others. This reframing can guide the development of new human-AI interactive tools, revise its uses in educational curricula, and inform ethical guidelines that safeguard the unique value of human creativity in the face of hyped AI technologies.

## Data Availability

The original contributions presented in the study are included in the article/supplementary material, further inquiries can be directed to the corresponding author.

## Footnotes

^1^We mention ChatGPT for clarity, but in this article we are concerned with LLMs in a conceptual, even Platonic, sense. We are not discussing specific instantiations such as ChatGPT, MidJourney, Stable Diffusion, or Claude.

^2^Barandiaran and Almendros’s (2024) prefer the term “generative” instead of “creative” to avoid the strong connotations of the latter.
